# Phytohormone and integrated mRNA and miRNA transcriptome analyses and differentiation of male between hermaphroditic floral buds of andromonoecious *Diospyros kaki* Thunb

**DOI:** 10.1186/s12864-021-07514-4

**Published:** 2021-03-23

**Authors:** Huawei Li, Liyuan Wang, Yini Mai, Weijuan Han, Yujing Suo, Songfeng Diao, Peng Sun, Jianmin Fu

**Affiliations:** 1 Key Laboratory of Non-timber Forest Germplasm Enhancement & Utilization of State Administration of Forestry and Grassland, No. 3 Weiwu Road, Jinshui District, Zhengzhou, 450003 China; 2 Non-timber Forest Research and Development Center, Chinese Academy of Forestry, No. 3 Weiwu Road, Jinshui District, Zhengzhou, 450003 China; 3 National Innovation Alliance of Persimmon Industry, No. 3 Weiwu Road, Jinshui District, Zhengzhou, 450003 China; 4 Research Institute of Forestry, Chinese Academy of Forestry, Beijing, 100091 China

**Keywords:** *Diospyros kaki*, Andromonoecy, Sex differentiation, Phytohormone, mRNA, miRNA

## Abstract

**Background:**

Persimmon (*Diospyros kaki* Thunb.) has various labile sex types, and studying its sex differentiation can improve breeding efficiency. However, studies on sexual regulation patterns in persimmon have focused mainly on monoecy and dioecy, whereas little research has been published on andromonoecy. In order to reveal the sex differentiation regulation mechanism of andromonoecious persimmon, we performed histological and cytological observations, evaluated *OGI* and *MeGI* expression and conducted phytohormones assays and mRNA and small RNA transcriptome analyses of the male and hermaphroditic floral buds of the andromonoecious persimmon ‘Longyanyeshi 1’.

**Results:**

Stages 2 and 4 were identified as the critical morphological periods for sex differentiation of ‘Longyanyeshi 1’ by histological and cytological observation. At both stages, *OGI* was differentially expressed in male and hermaphroditic buds, but *MeGI* was not. This was different from their expressions in dioecious and monoecious persimmons. Meantime, the results of phytohormones assays showed that high IAA, ABA, GA_3_, and JA levels at stage 2 may have promoted male floral bud differentiation. However, high JA levels at stage 4 and high ZT levels at stages 2 and 4 may have promoted hermaphroditic floral bud differentiation. In these phytohormone biosynthesis and signaling pathways, 52 and 54 differential expression genes (including *Aux/IAA*, *ARFs*, *DELLA*, *AHP*, *A-ARR*, *B-ARR*, *CYP735A*, *CRE1*, *PP2C*, *JAZ*, *MYC2*, *COI1*, *CTR1*, *SIMKK*, *ACO,* and *MPK6*) were identified, respectively. During the development of male floral buds, five metacaspases genes may have been involved in pistil abortion. In addition, *MYB*, *FAR1*, *bHLH, WRKY,* and *MADS* transcription factors might play important roles in persimmon floral bud sex differentiation. Noteworthy, miR169v_1, miR169e_3, miR319_1, and miR319 were predicted to contribute to phytohormone biosynthesis and signaling pathways and floral organogenesis and may also regulate floral bud sex differentiation**.**

**Conclusion:**

The present study revealed the differences in morphology and phytohormones content between male and hermaphroditic floral buds of ‘Longyanyeshi 1’ during the process of sex differentiation, and identified a subset of candidate genes and miRNAs putatively associated with its sex differentiation. These findings can provide a foundation for molecular regulatory mechanism researching on andromonoecious persimmon.

**Supplementary Information:**

The online version contains supplementary material available at 10.1186/s12864-021-07514-4.

## Background

Persimmon (*Diospyros kaki* Thunb.) is one of the important fruit species in China [1]. However, the persimmon industry has been affected by short fruiting periods, low shelf life, and transportation difficulties. Therefore, strengthening the cultivation of superior varieties is an important way to improve the development of the persimmon industry. Crossbreeding is an important means of germplasm innovation and thoroughbred breeding. However, there are no fruits in the male plant, and it is difficult to induce the conversion of male to female plant by artificial regulation in persimmon [2]. As a result, the selection of hybrid male parent with important economic traits is difficult, which limits the development of crossbreeding. Andromonoecy is the intermediate type of sexual system between the monoecious and dioecious type [3]. Andromonoecious persimmon can be used to study the regulation mechanism of sex differentiation, and these types of studies can serve as a guide for inducing the transition from male to andromonoecious plant and for cultivating hermaphroditic floral buds that bear fruits. This can also improve the efficiency of hybrid male parent selection and promote the development of crossbreeding.

Extensive research on sex differentiation in persimmon has been conducted in recent years. *Diospyros lotus* is a diploid and closely related to *D. kaki*. In the former, a microRNA encoded by the pseudogene *OGI* on the Y chromosome inhibits the expression of the autosomal transcription factor *MeGI* and male flowers development [4]. In hexaploid persimmons, *OGI* is nearly silenced by the insertion of ‘*kali’* into its promoter. DNA methylation level of the *MeGI* promoter determines *MeGI* expression and flower sex [5]. In the monoecious persimmon ‘Zenjimaru’, male and female flower development was divided into 11 stages progressing between June of one year and May of the following year. This process is characterized by key morphological periods in mid-June and the following mid-April [6]. High GA_3_ content is positively correlated with the formation of male floral buds, and high levels of ZT and ABA may promote the differentiation of female floral buds in persimmon [7].

Various phytohormones regulate flower development and sex differentiation. Gibberellins are usually considered to be masculinizing phytohormones, whereas ethylene generally has a feminizing effect [8]. Interactions between auxin and cytokinins determine flower types in several plant species [9]. Synergy between brassinolide and jasmonate inhibits tassel development in the male maize flower [10, 11]. Exogenous plant growth regulators or inhibitors alter the sex of *Cannabis sativa* [12], *Spinacia oleracea* [13], and *Carica papaya* [14].

MiRNAs participate in several regulatory pathways controlling plant reproductive development. MiR156 and miR172 are associated with *Arabidopsis* and maize flowering time [15, 16]. MiR172 regulates *Arabidopsis* flower development by targeting *APETALA2* [17]*.* In maize, *IDS1* translation is inhibited by *ts4* miRNA (miRNA172) and results in male florets. In contrast, a loss of-function mutation of *ts4* or a mutation of the *ids1* miRNA binding site produces normal IDS1 protein and results in the formation of female florets [18, 19].

**Fig. 1:**
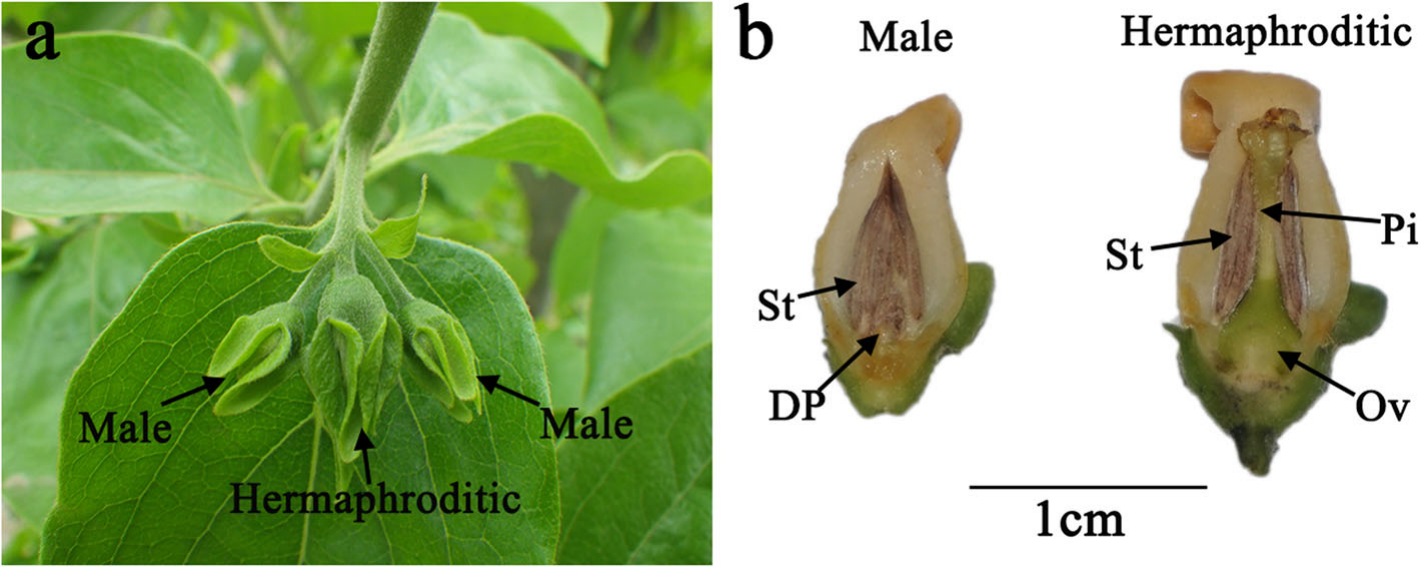
Male and hermaphroditic ‘Longyanyeshi 1’ floral buds. **a** Three-flower cyme. **b** Male and hermaphroditic floral bud anatomy. St: stamen; DP: defective pistil; Pi: pistil; Ov: ovary

Several previous studies have elucidated sex differentiation in monoecious and dioecious persimmons. Nevertheless, the sex regulation mechanism in the andromonoecious persimmon ‘Longyanyeshi 1’ (with hermaphroditic and male flowers) is unknown (Fig. 1) [20]. Here, the male and hermaphroditic floral buds of ‘Longyanyeshi 1’, which were in the critical morphological periods for sex differentiation, were used for phytohormones assays and mRNA and small RNA transcriptome analyses to identify the regulatory roles of phytohormones, candidate genes, and miRNAs in sex differentiation of andromonoecious persimmon. This study provides valuable information for further exploration of sex differentiation of the peculiar andromonoecious persimmon.

## Results

### Morphological comparison of male and hermaphroditic floral buds

**Fig. 2:**
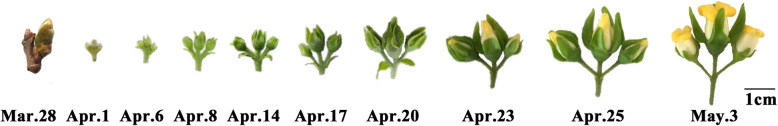
External morphological changes of the ‘Longyanyeshi 1’ floral buds

The bud scales of the ‘Longyanyeshi 1’ persimmon tree in Yuanyang County, Henan Province loosened and turned green on March 28. As the floral buds grew and developed, three-flower cymes were fully exposed by April 6. The floral bud sepals everted by April 14. Between April 14 and April 20, the floral buds expanded and grew but did not change in appearance. On April 23, the sepals opened and yellow-white petals appeared. The floral buds bloomed on May 3 (Fig. 2).

**Fig. 3:**
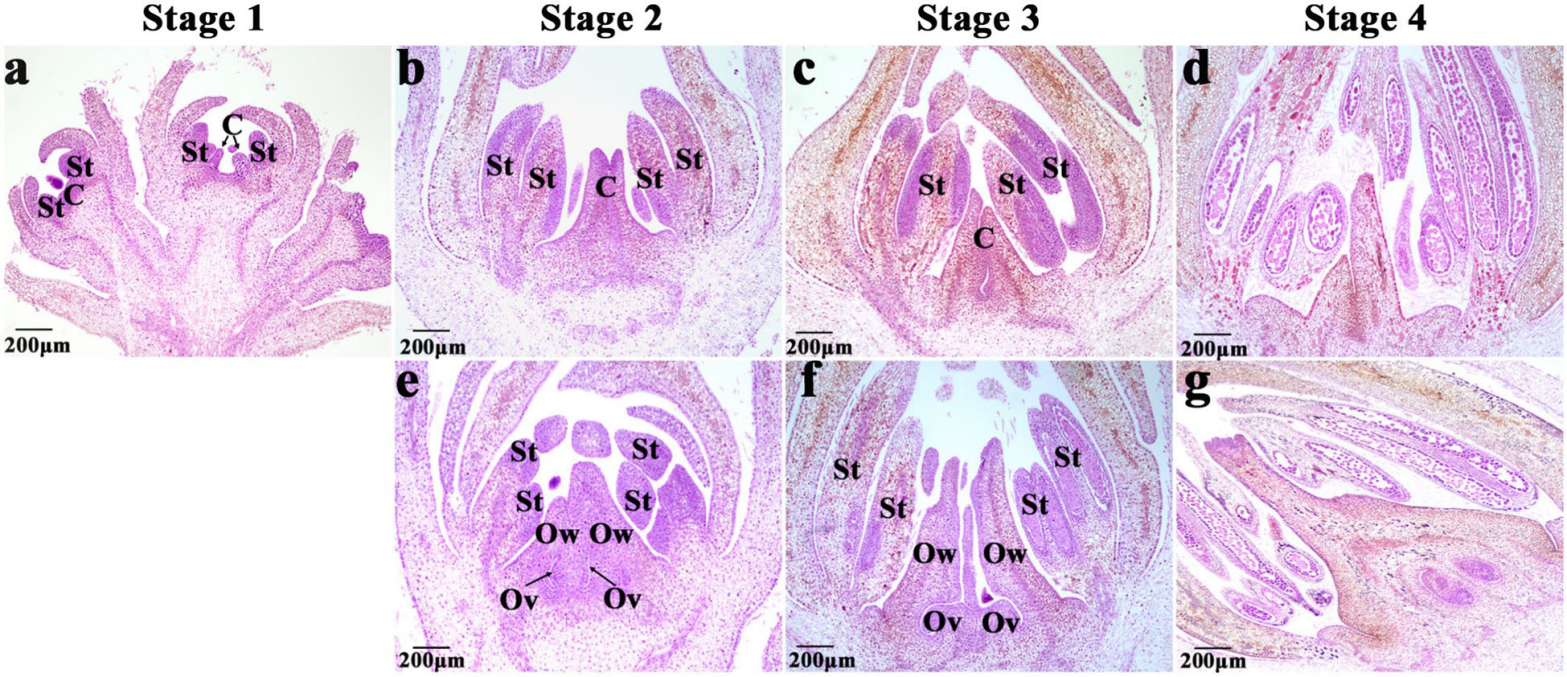
Internal morphological differences between male and hermaphroditic ‘Longyanyeshi 1’ persimmon floral buds. **a** Stamen and carpel primordia appeared in male and hermaphroditic floral buds. **b** Anther primordia appeared but neither ovaries nor ovules formed in the carpel bases. **c** Filament and anther compartments appeared. **d** Pistils were aborted. **e** Anther, style, stigma, ovary, and ovule primordia appeared. **f** Filament and anther compartments appeared and funicles formed. **g** Stamens and carpels developed normally. St: stamen; C: carpel; Ov: Ovule; Ow: ovary wall

Four representative stages were selected to describe the internal morphological differences between male and hermaphroditic floral buds. Stamen and carpel primordia were observed in the male and hermaphroditic floral buds at stage 1 (March 28–31) (Fig. 3a). At stage 2 (April 1–6), stamen primordia in the male floral buds differentiated into anther primordia and carpel primordia differentiated into styles and stigmata without basal ovaries or ovules (Fig. 3b). By stage 3 (April 8–10), anther primordia differentiated into filament and anther compartments and carpel primordia elongated slightly (Fig. 3c). After anther primordium differentiation during stage 4 (April 17–20), microspore mother cells entered meiosis and pistils stopped growing and were aborted (Fig. 3d). The stamen primordia at stage 2 of the hermaphroditic floral buds differentiated into anthers and carpel primordia differentiated into style, stigma, ovary, and ovule primordia (Fig. 3e). The stamen primordia at stage 3 differentiated into filament and anther compartments, and ovule primordia bulged and bent downwards to form basal funicles (Fig. 3f). By stage 4, the stamens and pistils were normally developed and were not aborted (Fig. 3g).

Stamen development in the male and hermaphroditic floral buds was synchronous. However, the fates of the pistils differed between the two sexual phenotypes. In the male floral buds, there were neither ovule or ovary primordia in the stage 2 carpels and the carpels were aborted by stage 4. In contrast, the carpels developed normally during the entire hermaphroditic floral bud development process. Hence, stages 2 and 4 are crucial morphological periods for sex differentiation in ‘Longyanyeshi 1’. We collected male and hermaphroditic floral buds at both of these stages for subsequent comparative analysis of their *OGI* and *MeGI,* endogenous phytohormone levels and mRNA and small RNA transcriptome expression levels.

### Differential expression analysis of *OGI* and *MeGI*

**Fig. 4:**
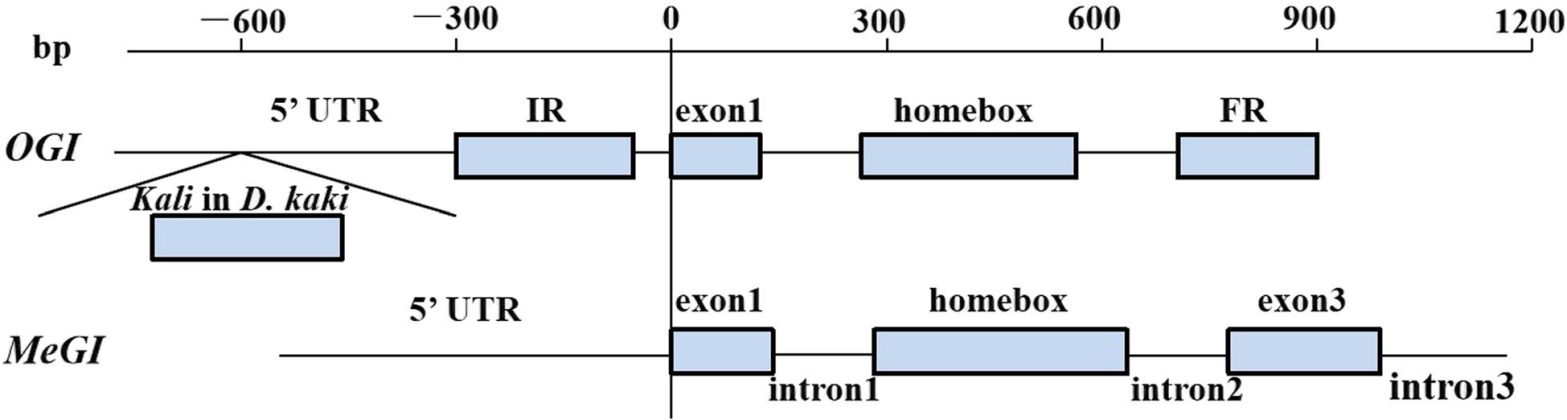
*OGI* and *MeGI* construction diagram. UTR: untranslated regions; IR: inverted repeat; FR: forward repeat

**Fig. 5:**
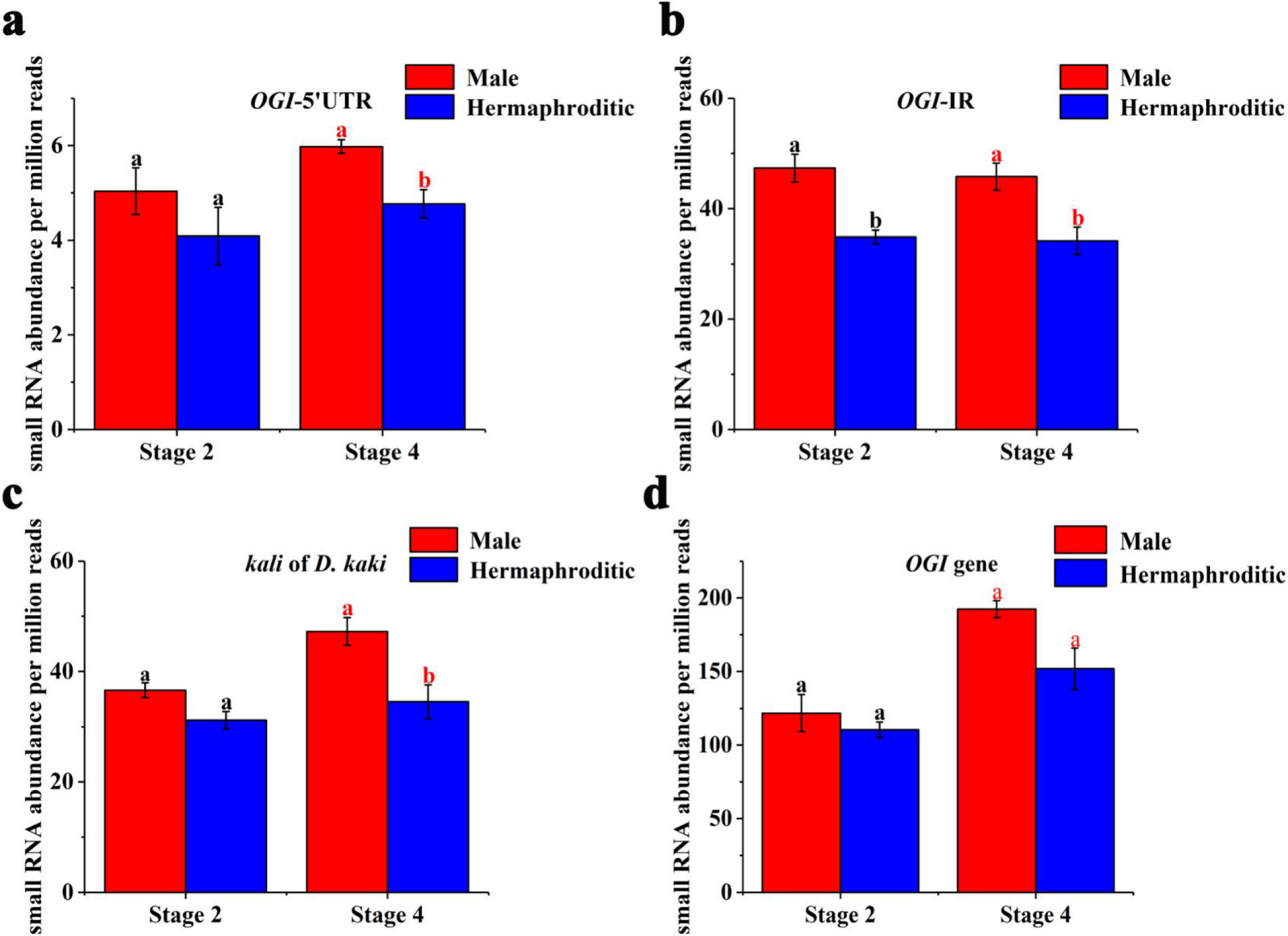
Small RNAs accumulation on the *OGI* genomic sequence*.* Data are expressed as the mean ± standard error of three replications. Red and black letters indicate a significant difference between male and hermaphroditic floral buds at each developmental stage, based on an independent T-test at the *P* < 0.05 significance level

**Fig. 6:**
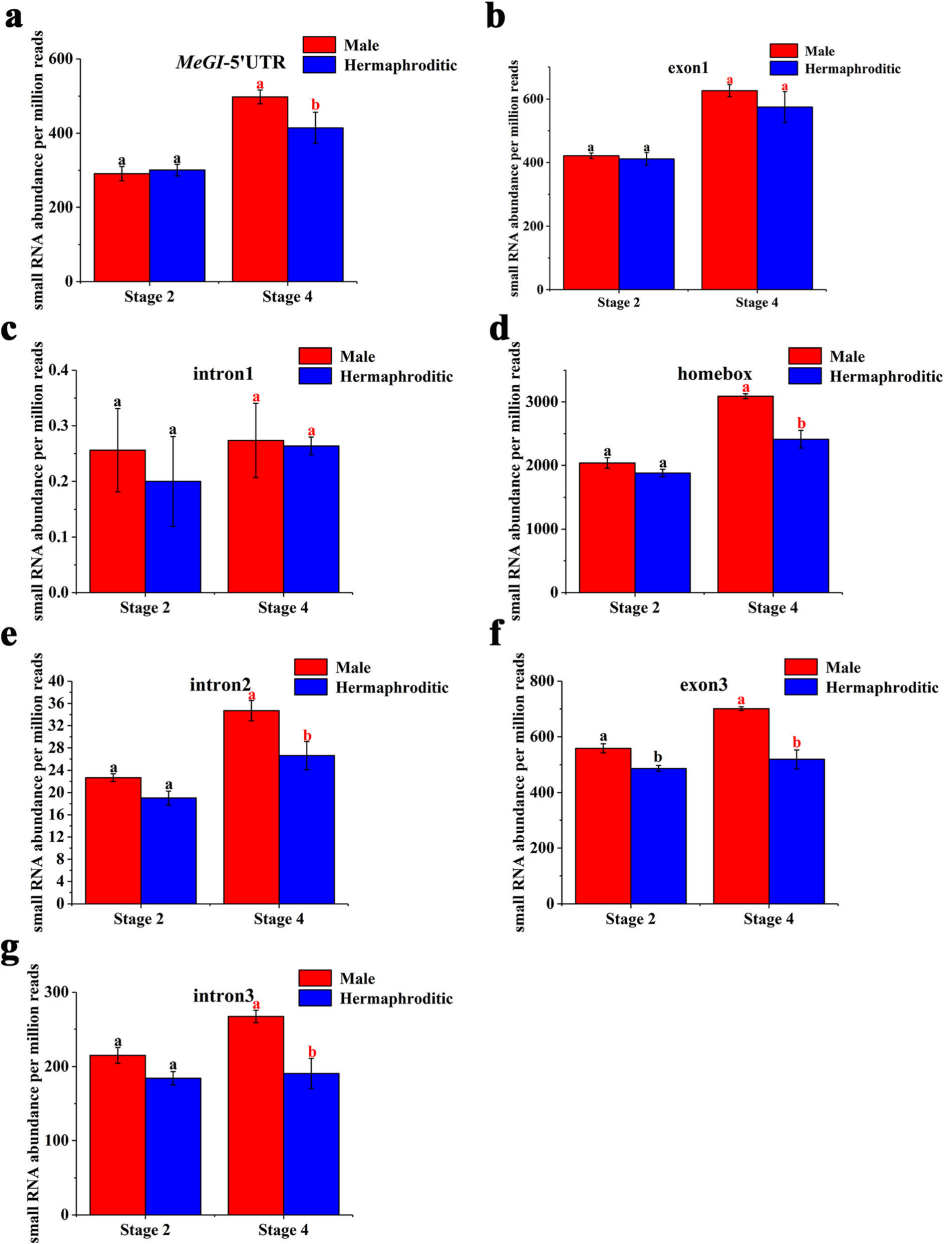
Small RNA accumulation on the *MeGI* genomic sequence. Data are expressed as the mean ± standard error of three replications. Red and black letters indicate a significant difference between male and hermaphroditic floral buds at each developmental stage, based on an independent T-test at the *P* < 0.05 significance level

**Fig. 7:**
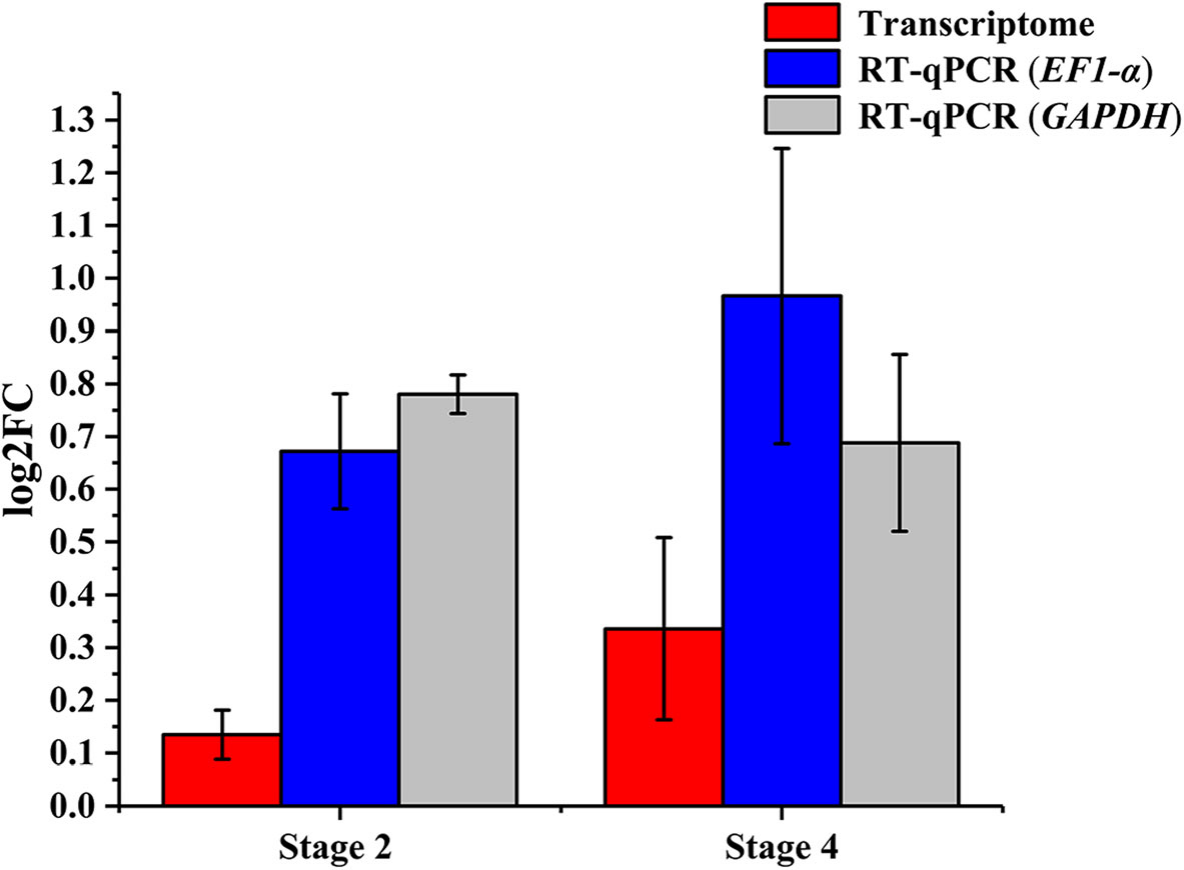
Differential expression of *MeGI.* FC, fold change

*OGI* is a pseudogene encoding only small RNA. It is highly homologous to *MeGI*. To estimate the expression levels of *OGI* in the male and hermaphroditic floral buds of andromonoecious persimmon, we calculated the accumulation levels of small RNAs on the *OGI* and *MeGI* genomic sequences in both floral bud types (Fig. 4). The results showed that small RNA accumulation levels in the *OGI* and *MeGI* genomic sequences of male floral buds were higher than those for the hermaphroditic floral buds at stages 2 and 4 (Fig. 5; Fig. 6). Thus, the expression levels of *OGI* in the male floral buds were higher than those in the hermaphroditic floral buds at these stages. However, there were no differences between male and hermaphroditic floral buds in terms of their *MeGI* expression levels at these stages according to the transcriptome and RT-qPCR analyses (Fig. 7).

### Phytohormone content in male and hermaphroditic floral buds

**Fig. 8:**
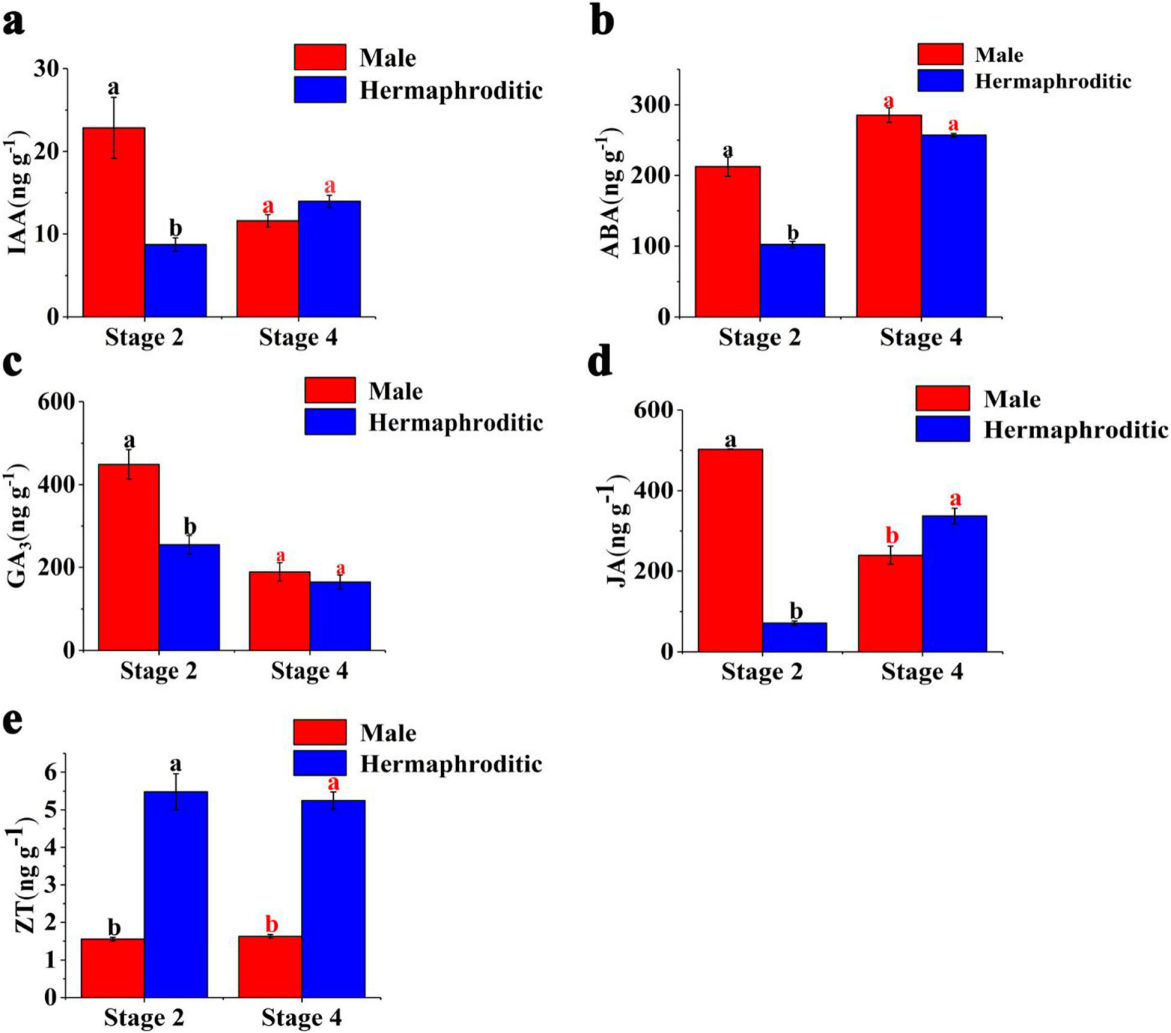
Phytohormone levels in male and hermaphroditic floral buds at various stages. **a** Indole-3-acetic acid (IAA). **b** Abscisic acid (ABA). **c** Gibberellin 3 (GA_3_). **d** Jasmonic acid (JA). **e** Zeatin (ZT). Data are expressed as the mean ± standard error of three replications. Red and black letters indicate a significant difference between male and hermaphroditic floral buds at each developmental stage, based on an independent T-test at the *P* < 0.05 significance level

To establish the effects of endogenous phytohormones on floral bud sex differentiation, we measured indole-3-acetic acid (IAA), abscisic acid (ABA), gibberellin 3 (GA_3_), jasmonic acid (JA), and zeatin (ZT) in male hermaphroditic floral buds at stages 2 and 4. The IAA, ABA, and GA_3_ levels in the male floral buds were markedly higher than those in the hermaphroditic floral buds at stage 2. However, there were no substantial differences between the two floral bud sexes in terms of their phytohormone levels at stage 4. JA level was higher in the male floral buds than it was in the hermaphroditic floral buds at stage 2. However, the opposite was true for stage 4. ZT levels were ~ 3.5-fold and ~ 3.2-fold higher in the hermaphroditic floral buds than in the male floral buds at stages 2 and 4, respectively (Fig. 8).

### Transcriptome sequencing

To identify the mRNA expression profiles in male and hermaphroditic floral buds, we constructed 12 cDNA libraries at stages 2 (MA1, MA2, and MA3 for the male and HA1, HA2, and HA3 for the hermaphroditic) and 4 (MB1, MB2, and MB3 for the male and HB1, HB2, and HB3 for the hermaphroditic) using total RNA and sequenced them on the BGISEQ-500 platform. A total of 44.42, 44.04, 44.32, 44.41, 42.65, 44.05, 44.40, 44.20, 42.75, 45.88, 44.00, and 42.47 Mb clean reads, respectively, were obtained after eliminating low-quality reads (Additional file 1: Table S1). Quality control was performed and the clean reads of the 12 libraries were assembled into 82,910 unigenes with an average length of 1376 bp (Additional file 2: Table S2). Among these, 22,768 unigenes were 200–500 bp long, 17,447 unigenes were 500–1000 bp long, and 42,695 unigenes were < 1000 bp long (Additional file 3: Fig. S1).

The assembled unigenes were annotated via BLAST in seven public databases (NR, NT, Swissprot, Kyoto Encyclopedia of Genes and Genomes (KEGG), KOG, Pfam, and Gene Ontology (GO)) and 62,021 (74.81%), 50,224 (60.58%), 46,639 (56.25%), 49,790 (60.05%), 49,614 (59.84%), 47,121 (56.83%), and 34,735 (41.89%) were aligned, respectively. A total of 64,355 unigenes accounting for 77.62% of the total were annotated in ≥1 public database (Additional file 4: Table S3).

**Fig. 9:**
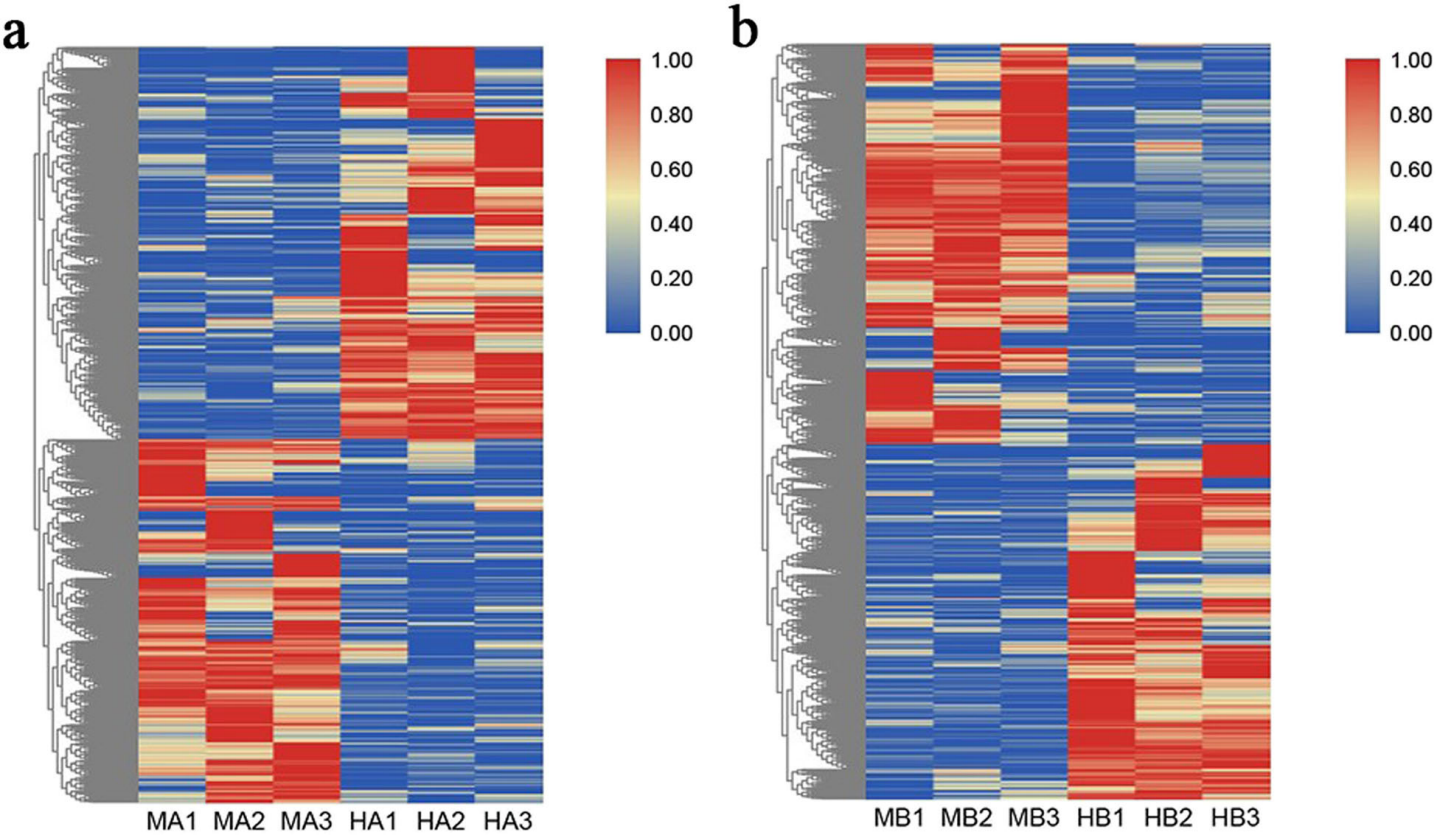
Heat map of clustered DEGs at stages 2 (**a**) and 4 (**b**). The original expression values of the DEG FPKM (fragments per kilobase per million) were normalized by Z-score

Here, 3684 DEGs were identified between the male and hermaphroditic floral buds. Compared with the hermaphroditic floral buds, 790 genes were upregulated and 855 were downregulated in the male floral buds at stage 2 and 1341 genes were upregulated and 1185 were downregulated at stage 4 (Fig. 9).

**Fig. 10:**
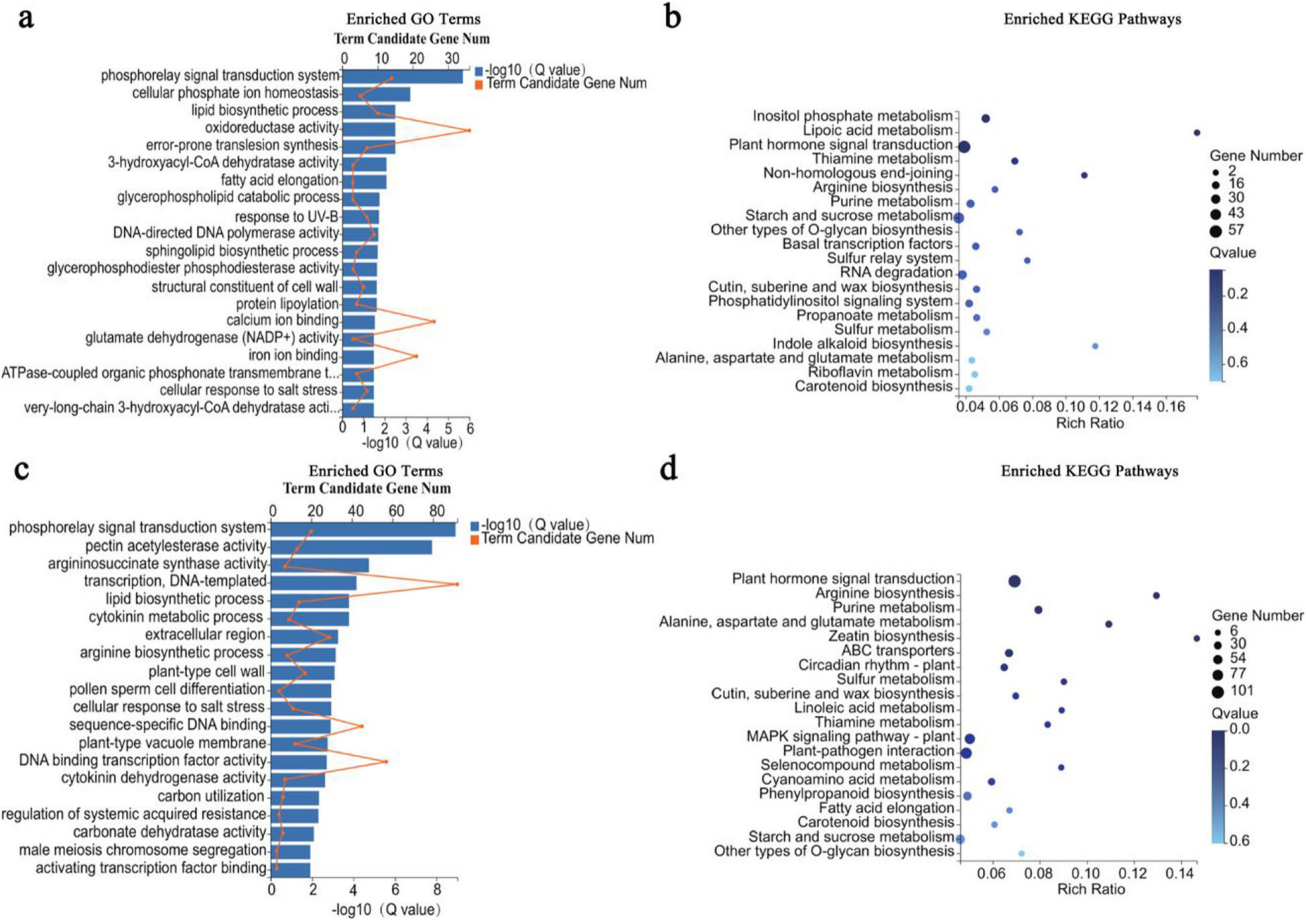
GO and KEGG pathway enrichment analyses of DEGs at stages 2 (**a**, **b**) and 4 (**c**, **d**)

A GO analysis of the DEGs between the male and hermaphroditic floral buds at stage 2 disclosed enrichment in 20 categories. The most abundant GO categories were oxidoreductase activity (GO:0016491; 36 unigenes), calcium ion binding (GO:0005509; 26), and iron ion binding (GO:0005506; 21) (Fig. 10a). The most highly enriched KEGG pathways in the male floral buds compared with the hermaphroditic floral buds were plant hormone signal transduction (57 unigenes), starch and sucrose metabolism (45), and RNA degradation (28) (Fig. 10b). For stage 4, the DEGs were classified by GO analysis into 20 categories. The most abundant were transcription, DNA-templated (GO:0006351; 92 unigenes), DNA binding transcription factor activity (GO:0003700; 57), and sequence-specific DNA binding (GO:0043565; 45) (Fig. 10c). Enriched KEGG pathways of the DEGs were plant hormone signal transduction (101 unigenes), plant-pathogen interaction (82), and MAPK signaling pathway-plant (70) (Fig. 10d).

### Identification of differentially expressed transcription factors

**Fig. 11:**
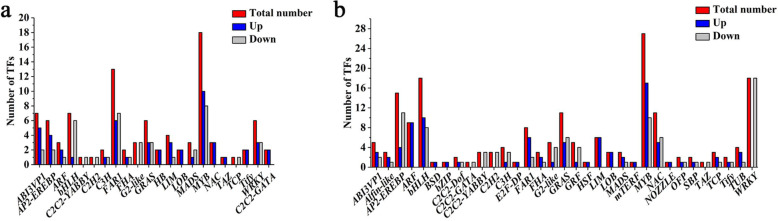
Numbers of differentially expressed TFs at stages 2 (**a**) and 4 (**b**)

Ninety-five transcription factors (TFs) belonging to 22 TF families and 183 TFs belonging to 33 TF families were differentially expressed at stages 2 and 4, respectively (Additional file 5: Table S4). *MYB*, *FAR1,* and *bHLH* were enriched at stage 2 and *MYB*, *bHLH,* and *WRKY* were enriched at stage 4. Fifty-two TFs were upregulated and 43 were downregulated in male floral buds at stage 2. Ninety-three TFs were upregulated and 91 were downregulated in male floral buds at stage 4 (Fig. 11).

### DEGs related to phytohormone biosynthesis and signaling pathways

**Fig. 12:**
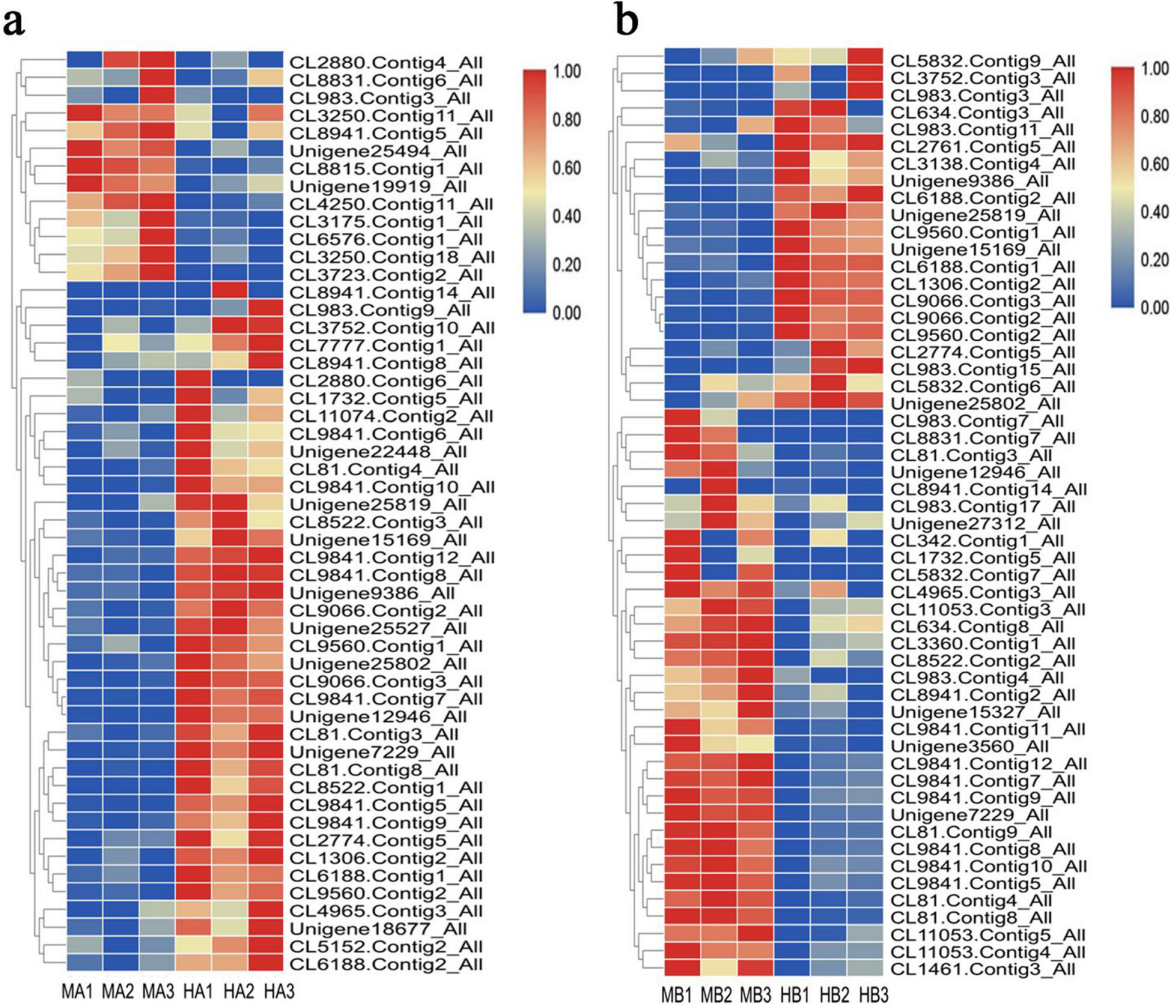
Heat map of DEGs related to phytohormone biosynthesis and signaling pathways at stages 2 (**a**) and 4 (**b**). The original expression values of the DEG FPKM (fragments per kilobase per million) were normalized by Z-score

The combining of the phytohormone and transcriptome analyses revealed 52 and 54 DEGs related to phytohormone biosynthesis and signaling pathways at stages 2 and 4, respectively. In the male floral buds, 13 DEGs were upregulated and 39 DEGs were downregulated at stage 2, whereas 33 DEGs were upregulated and 21 DEGs were downregulated at stage 4 (Additional file 6: Table S5). The expression levels of the DEGs at stages 2 and 4 are depicted in a heat map (Fig. 12).

In the auxin signaling pathways, the *AUX/IAA* genes and one of the *ARFs* (CL2880.Contig4_All) were upregulated, whereas the other *ARF* (CL2880.Contig6_All) was downregulated in the male floral buds at stage 2.

In the gibberellin signaling pathways, the *DELLA* genes and one *TF* (CL4250.Contig11_All) were highly upregulated in the male floral buds at stage 2.

In the cytokinin biosynthesis and signaling pathways, the *AHP*, *A-ARR,* and *CYP735A* genes were downregulated in the male floral buds at stage 2. The *CRE1* and *AHP* genes were upregulated and the *A-ARR* and *CYP735A* genes were downregulated in the male floral buds at stage 4.

In the abscisic acid signaling pathways, the *PP2C* gene was upregulated in the male floral buds at stage 2.

In the jasmonate signaling pathways, the *JAZ* and *MYC2* genes were upregulated and downregulated, respectively, in the male floral buds at stage 2. The *COI1* and one *JAZ* (CL634.Contig3_All) genes were downregulated in the male floral buds at stage 4.

Ethylene is important for sex differentiation in plants. Here, the DEGs associated with ethylene biosynthesis and signaling pathways were also identified. The *CTR1*, *SIMKK,* and *ACO* genes were downregulated in the male floral buds at stage 2, whereas the *CTR1* and *MPK6* genes were upregulated and the *ACO* genes were downregulated in the male floral buds at stage 4.

### DEGs related to programmed cell death

Programmed cell death (PCD) is responsible for the abortion of inappropriate sex organs in persimmon floral buds and leads to the formation of unisexual flowers [21]. Five DEGs belonging to the metacaspase family were identified at stage 4. Among these, three were upregulated and two were downregulated in the male floral buds (Table 1).

**Table 1:** DEGs related to PCD at stage 4

Genes ID	log2FC (MA/HA)	Q value	Nr Description
CL10736.Contig5_All	2.180758613	0.00082578	Metacaspase-1-like
CL1552.Contig4_All	2.456003575	2.35E-06	Metacaspase-1-like
CL1552.Contig9_All	−4.740082502	0.000780007	Putative metacaspase family protein
CL1552.Contig12_All	−2.282189273	1.81E-53	Metacaspase-1-like
CL1552.Contig16_All	1.495553765	1.39E-05	Metacaspase-1-like

### MiRNA sequencing

**Fig. 13:**
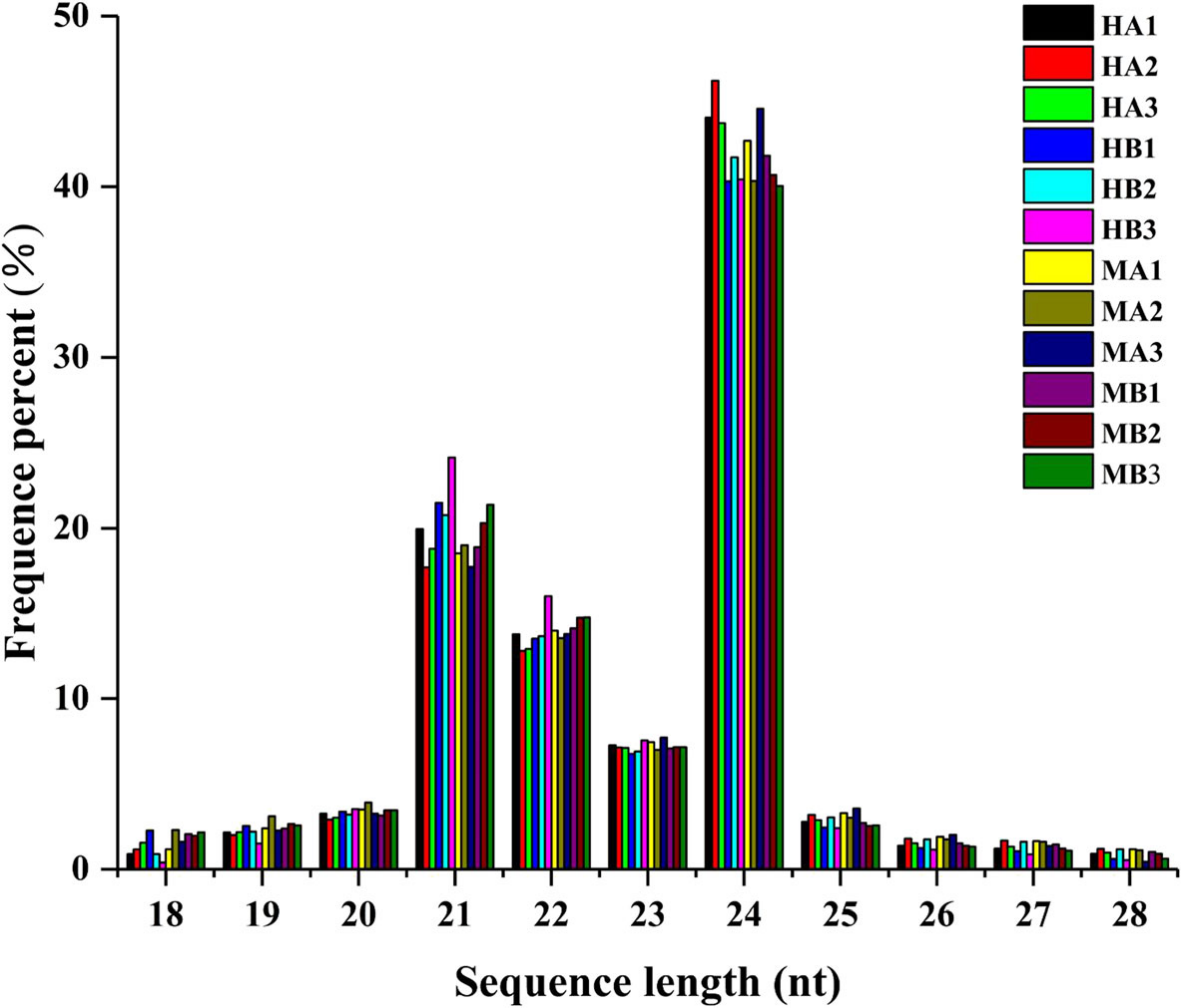
Small RNA length distribution and abundance in 12 libraries. X-axis, length of small RNA distribution; Y-axis, corresponding percentage of raw reads

Twelve small RNA libraries at stages 2 (MA1, MA2, and MA3 for the male and HA1, HA2, and HA3 for the hermaphroditic) and 4 (MB1, MB2, and MB3 for the male and HB1, HB2, and HB3 for the hermaphroditic) were constructed with total RNA and sequenced on the BGISEQ-500 platform to investigate miRNA differentiation between the male and hermaphroditic floral buds. After removing invalid adapter and low-quality sequences, 28,031,142, 28,702,111, 27,811,471, 27,071,649, 28,343,565, 28,194,623, 27,935,952, 27,231,677, 27,100,665, 28,377,409, 28,193,129, and 27,702,413 clean reads with lengths of 18–30 nt were generated (Additional file 7: Table S6). The miRNA length distributions were similar among libraries, and 24-nt RNAs were the most abundant (Fig. 13). Fifty-two conserved miRNAs in 20 miRNA families and 81 predicted novel miRNAs were found in the 12 small RNA libraries (Additional file 8: Table S7).

We compared the miRNA expression between the male and hermaphroditic floral buds at stages 2 and 4 in order to detect differentially expressed miRNA (DEMs). Nineteen differentially expressed miRNAs (including 12 conserved and seven novel miRNAs) were identified. Compared with the hermaphroditic floral buds at stage 2, 7 DEMs were upregulated and 7 were downregulated in the male floral buds. At stage 4, 3 DEMs were upregulated and seven were downregulated in the male floral buds (Table 2).

**Table 2:** DEMs at stages 2 and 4

Stage	miRNA name	miRNA sequence	log2Ratio (MA/HA)	Q value
Stage 2	miR157a-5p	TTGACAGAAGATAGAGAGCAC	−1.292327831	0
	miR157d	TGACAGAAGATAGAGAGCAC	−1.611290674	5.20E-10
	miR157d-3p	GCTCTCTATGCTTCTGTCATC	−1.16042311	0
	miR166e	GGACCAGGCTTCATTCCCC	2.536704288	0
	miR169b-5p	CAGCCAAGGATGACTTGCCGG	1.126376469	2.38E-125
	miR169e_3	AGCCAAGGATGACTTGCCGG	−5.573054385	0
	miR169v_1	CAGCCAAGGATGACTTGCC	5.253333465	0
	miR319_1	TTGGACTGAAGGGAGCTCC	2.431345847	2.35E-185
	miR319a	CTTGGACTGAAGGGAGCTCC	−3.391424127	1.93E-161
	miR390e	AGCTCAGGAGGGATAGCGCC	−1.188527882	1.25E-23
	novel_mir27	GCTTTACTTCGGAGCGGGAGTACTCGA	−4.45853832	0.00051746
	novel_mir30	CATCGGTGGCCATAGAAAAGGAAAAAG	4.585855796	0.00028378
	novel_mir35	TAAGGTGAAAGGTGTGTGTTT	4.701333014	0.00015746
	novel_mir63	AATTTAATATCATGTGGGCCGATG	4.701333014	0.00015746
Stage 4	miR166e	GGACCAGGCTTCATTCCCC	−1.397911379	0
	miR166m_2	CGGACCAGGCTTCATTCCCC	1.361643651	0
	miR169e_3	AGCCAAGGATGACTTGCCGG	−6.747470965	0
	miR169v_1	CAGCCAAGGATGACTTGCC	7.035071502	0
	miR171a_3	TGATTGAGCCGTGCCAATAT	−1.01935132	1.56E-24
	miR319a	CTTGGACTGAAGGGAGCTCC	−1.190745132	7.98E-26
	miR390e	AGCTCAGGAGGGATAGCGCC	−1.465393915	6.28E-46
	novel_mir1	GTGGATTGGACATTTAGTTTGC	−4.57059320	0.0005949
	novel_mir25	CACAATGACACGCCAACGGCGCA	1.146226258	1.55E-53
	novel_mir46	AACCCATTGATTCCCAAATTT	−5.509192658	1.02E-06

### DEG and DEM correlations

To clarify the functional miRNA-mRNA interactions, we sought cognate mRNA targets for the DEMs and their predicted target lists using psRobot, TAPIR, and TargetFinder. There were nine and five miRNA-mRNA pairs at stages 2 and 4, respectively (Table 3).

**Table 3:** MiRNA-mRNA pairs at stages 2 and 4

Stage	miRNA	Target gene	log2FC (MA/HA)	Nr Description
Stage 2	miR157d	CL2363.Contig6_All	-1.262814541	Vacuolar amino acid transporter 1
	miR157d	Unigene22044_All	-5.102609588	Hypothetical protein VITISV_025837
	miR166e	CL1331.Contig8_All	1.397213153	Phabulosa
	miR169e_3	CL1032.Contig1_All	1.094476454	Nuclear transcription factor Y subunit A-1-like isoform X1
	miR169e_3	CL1314.Contig2_All	6.041229196	Serine/threonine-protein kinase ppk15 isoform X2
	miR169v_1	CL1032.Contig1_All	1.094476454	Nuclear transcription factor Y subunit A-1-like isoform X1
	miR169v_1	Unigene33268_All	-2.028567844	26S proteasome non-ATPase regulatory subunit 2 homolog A
	miR319_1	CL1678.Contig7_All	-3.102755082	Transcription factor TCP2
	miR319a	CL1678.Contig7_All	-3.102755082	Transcription factor TCP2
Stage 4	miR169e_3	CL1314.Contig19_All	1.300124884	Probable serine/threonine-protein kinase dyrk1
	miR169e_3	CL1314.Contig20_All	-1.990939687	Dual specificity protein kinase CLK1 isoform X1
	miR319a	CL1678.Contig7_All	2.61840453	Transcription factor TCP2
	miR390e	CL3825.Contig3_All	2.120159009	Probable LRR receptor-like serine/threonine-protein kinase At1g63430
	novel_mir1	CL1148.Contig10_All	-1.354299186	Pheophytinase, chloroplastic isoform X2

### DEG and DEM validation by RT-qPCR

**Fig. 14:**
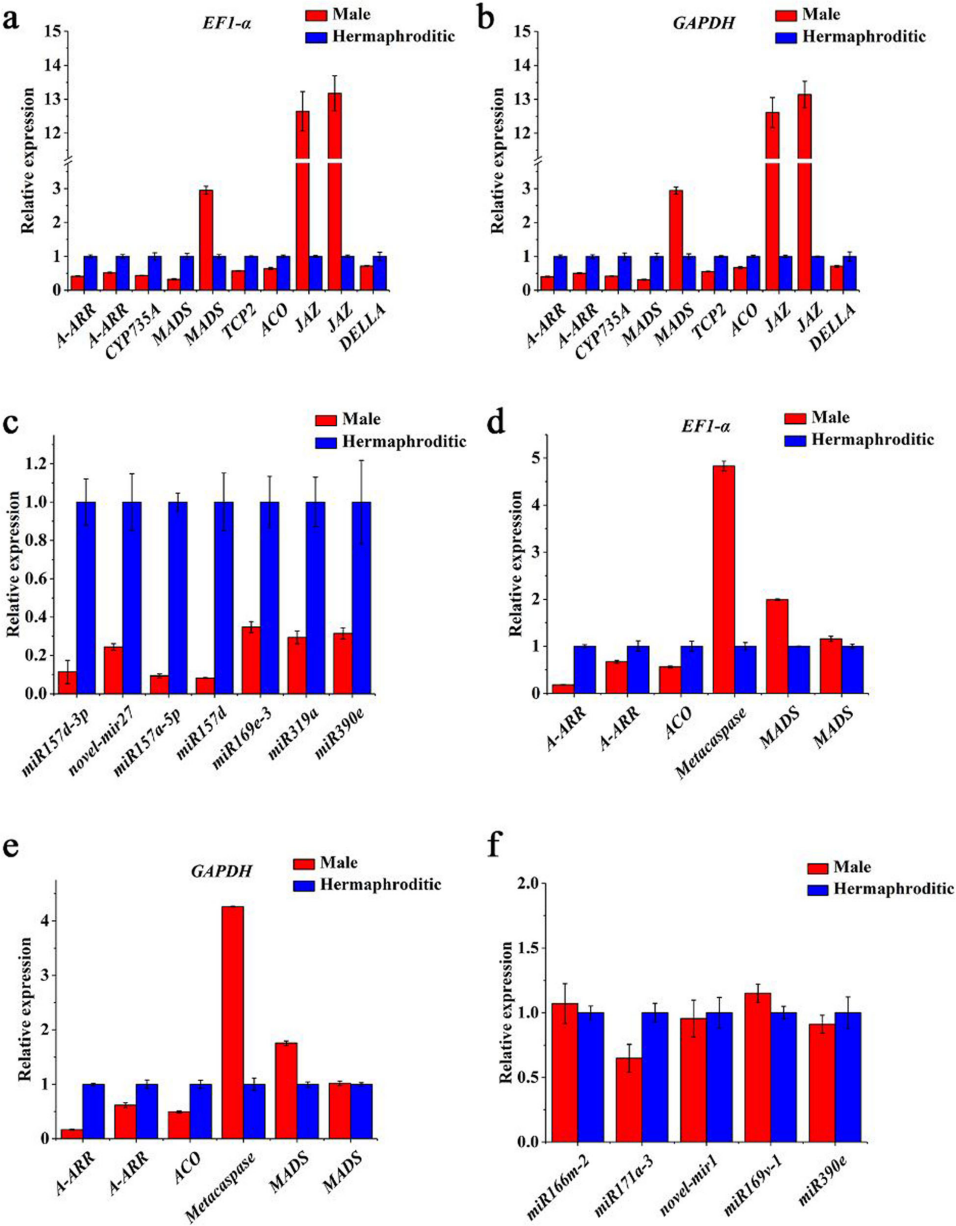
DEG and DEM validation at stages 2 (**a**, **b**, **c**) and 4 (**d**, **e**, **f**) by RT-qPCR

Ten DEGs and seven DEMs at stage 2 and six DEGs and five DEMs at stage 4 were selected to validate transcriptome data accuracy by RT-qPCR. The expression patterns of these genes and their miRNAs were consistent with the RNA-seq and small RNA-seq results. Thus, our sequencing data were reliable (Fig. 14).

## Discussion

Unisexual flowers evolved from hermaphroditic flowers [22]. However, the development process of unisexual flowers is different among different plant species. In some species, the flowers are initially hermaphroditic, and subsequent present with degeneration of the pistil or stamen primordia results in the formation of unisexual flowers. This mechanism was observed in *Silene latifolia* [23] and *D. kaki* cultivar ‘Zenjimaru’ [6]. In another development process, sex differentiation of pistils and carpels proceeds normally and there is no abortion of inappropriate sex organs. This mechanism was observed in *Cannabis sativa* and *Populus deltoides* [24]. Here, all floral buds on both sides of the three-flower cyme of ‘Longyanyeshi 1’ persimmon underwent an initial hermaphroditic period. The pistil primordia of the lateral two floral buds were then arrested and formed male flowers during the microspore mother cell period. In contrast, the pistil and stamen of the central flowers developed normally and formed hermaphroditic flowers. The critical time point for pistil primordia abortion in the male flowers of andromonoecious persimmon coincided with that of a monoecious persimmon reported in a previous study [6].

*OGI* and *MeGI* are the key regulators of persimmon sex differentiation. *OGI* and *MeGI* promoter methylations regulate *MeGI* expression in androecious *D. lotus* and monoecious *D. kaki*, respectively. High expression of *MeGI* promotes female flower development and inhibits male flower development in persimmon [4, 5]. In this study, the expression levels of *OGI* at stages 2 and 4 were higher in the male floral buds than they were in the hermaphroditic floral buds. However, there were no significant differences between the male and hermaphroditic floral buds at stages 2 and 4 in terms of their *MeGI* expression levels. Thus, in addition to the *OGI/MeGI* system, novel regulatory mechanisms may be underlying sex differentiation in andromonoecious persimmon in addition to the *OGI/MeGI* system. Thus, we performed phytohormone assays and mRNA and small RNA transcriptome analyses of male and hermaphroditic floral buds of the andromonoecious persimmon ‘Longyanyeshi 1’.

Phytohormones are important flower development regulators in many plant species [4] and their effects on sex differentiation are widely known [25]. Cytokinins promote female flower development in certain plant species. Exogenous cytokinins transform male *Vitis* spp. flowers into hermaphroditic flowers [26]. Genotypically male mercury flowers acquire feminine characteristics after cytokinin treatment [27]. In *Actinidia* spp*.*, exogenous cytokinin application partially restored gynoecium development in male flowers [28]. In this study, the ZT levels at stage 2 and 4 were higher in hermaphroditic floral buds than in male floral buds, and *AHP*, *A-ARR*, *B-ARR*, *CYP735A*, and *CRE1* genes, which are involved in cytokinin biosynthesis and signaling for regulating flower growth and development [29–35], were differentially expressed at both stages, which indicated that high ZT levels can promote the development of hermaphroditic floral buds at stage 2 and 4, and that these genes may offer excellent candidates in the sex differentiation of ‘Longyanyeshi 1’.

Jasmonate can participate in root growth, senescence, and reproductive organ formation [36]. Previous studies have shown that jasmonate determines male identity in maize tassel [37]. Mutants defective in jasmonate synthesis or signaling transduction are male-sterile [38]. In this study, the JA levels at stage 2 were higher in male floral buds than in hermaphroditic floral buds, but the opposite trend was observed at stage 4. Furthermore, *JAZ*, *COI1*, and *MYC2* genes, which play a central role in JA signaling [39, 40], were differentially expressed at stage 2 or 4, suggesting that JA played different roles in andromonoecious persimmon sex differentiation at different development stages, and that high JA levels were beneficial to the development of male floral buds at stage 2, whereas they were more beneficial to the development of hermaphroditic floral buds at stage 4. And JA signaling transduction genes may have important roles in andromonoecious persimmon sex differentiation.

Auxin masculinizes numerous plant species, such as *Humulus lupulus* [41] and *Mercurialis annua* [42]. Our results were in accordance with previous reports that the IAA levels at stage 2 were higher in male floral buds than in hermaphroditic floral buds, which indicated that high IAA levels were contributed to the formation of male floral buds at this stage. Aux/IAA are early auxin response proteins that participate in auxin signaling by interacting with ARF proteins as transcriptional repressors [43]. Among auxin signaling-related genes, *Aux/IAA* and *ARFs* were highly expressed in male floral buds at stage 2, suggesting that these genes may be important candidates in the sex differentiation of ‘Longyanyeshi 1’.

Gibberellins influence male expression in different plant species [44, 45]. The application of gibberellin inhibitors induces pistil development in male papaya [24]. In this study, the GA_3_ levels at stage 2 were higher in male floral buds than in hermaphroditic floral buds, Furthermore, *DELLA* genes, which are nucleus-localized transcription regulators that participate in GA signaling and flower development [46], were identified at this stage, indicating that high GA_3_ levels were essential for the development of male floral buds at stage 2, and *DELLA* genes may participate in the GA signaling to regulate the sex differentiation of ‘Longyanyeshi 1’.

Abscisic acid regulates multiple flower development processes [45] including initiation, differentiation, and senescence. In this study, the ABA levels at stage 2 were higher in male floral buds than in hermaphroditic floral buds, which indicated that high ABA levels may play important roles in the development of male floral buds at this stage. *PP2C* has a key role in ABA signaling, and it was upregulated in male floral buds at stage 2, which was inconsistent with the result of a previous study that showed that *PP2C* is a negative regulator of ABA responses [47].

Ethylene is thought to be vital for plant sex differentiation [48]. It has a major role in female phenotypic expression [8]. The *ACO* genes are critical for ethylene biosynthesis [49]. Here, *ACO* was upregulated in the hermaphroditic floral buds at stages 2 and 4. Therefore, ethylene may promote pistil development in hermaphroditic ‘Longyanyeshi 1’ floral buds.

TFs are crucial for gene networks. Members of the MYB protein family are flower development regulators [50], and certain *MYB* TFs are DELLA-responsible GA response genes involved in stamen and pollen maturation [51]. The *bHLH* and *FAR1* TFs participate in light signaling and flowering timing regulation in *Arabidopsis* [52, 53]. The *WRKY* TFs have various roles in plant disease resistance, abiotic stress response, nutrient deprivation, senescence, and phytohormone-controlled processes [54]. The transcription factor MADS controls sex differentiation by regulating reproductive organ development in plants [55–59]. In this study, 18 *MYB*, 13 *FAR1,* 7 *bHLH*, and 3 *MADS* TFs were differentially expressed at stage 2, and 27 *MYB*, 18 *bHLH*, 18 *WRKY*, and 3 *MADS* TFs were differentially expressed at stage 4. These TFs may have performed certain sex differentiation-related functions in ‘Longyanyeshi 1’ floral buds.

Programmed cell death (PCD) is a normal part of a multicellular organism’s life. It is active and orderly and may occur at various developmental stages. In ‘Zenjimaru’ (*D. kaki*), abortion of inappropriate pistil and stamen primordia was closely associated with PCD [21]. Members of the metacaspase family might induce PCD [60]. Here, five genes belonging to the metacaspase family were identified at stage 4. Metacaspases might be implicated in male floral bud development, as they suppress pistil primordia.

MiRNAs regulate various biological and metabolic processes associated with plant growth and development such as signal transduction and responses to biotic and abiotic stress [61]. In our study, miR169v_1 and miR169e_3 were significantly upregulated and downregulated, respectively, in male floral buds at stage 2. Their target nuclear transcription factor Y subunit A-1-like isoform X1 (*NFYA1*), which performs redundant roles in male gametophyte development, embryogenesis, seed development, and post-germinative growth [62], was upregulated at this stage. The 26S proteasome non-ATPase regulatory subunit 2 homolog A (*RPN1A*), which is the target of miR169v_1 and downregulates ABA signaling transduction [63], was markedly downregulated at this stage. Thus, miR169v_1 plays an important part in male floral bud development at stage 2 as it downregulates *RPN1A* and controls phytohormone biosynthesis. Both miR169v_1 and miR169e_3 have key functions in the regulation of sex differentiation in andromonoecious persimmon because they target *RPN1A* or *NFYA1.* Here, miR319_1 was downregulated in the hermaphroditic floral buds at stage 2, whereas miR319a was upregulated in them at stages 2 and 4. Their target transcription factor TCP2 (*TCP2*) is involved in embryonic growth, floral organogenesis, pollen development, diurnal rhythm, and phytohormone signal transduction [64]. *TCP2* was upregulated at stage 2 and downregulated at stage 4 in the hermaphroditic floral buds. As miR319_1 and miR319a target *TCP2*, they may regulate floral bud sex differentiation in andromonoecious persimmon.

The current results suggest that exogenous phytohormone spraying may be used to artificially regulate the conversion of male flowers to hermaphroditic flowers in persimmon, which will be valuable in crossbreeding. In addition, the selected candidate genes and miRNAs can be used for subsequent functional verification and construction of a molecular regulatory network for sex differentiation of andromonoecious persimmon.

## Conclusion

To our knowledge, the present study is the first to describe the sex differentiation mechanism in the floral buds of the andromonoecious persimmon ‘Longyanyeshi 1’. The absence of ovary primordia and ovules in the carpels at stage 2 and the abortion of carpels at stage 4 distinguished the male from the hermaphroditic floral buds. Thus, stages 2 and 4 are critical morphological periods for sex differentiation of ‘Longyanyeshi 1’. Differential expression analysis of *OGI* and *MeGI* showed that a novel supplementary mechanism may determine andromonoecious persimmon sexuality. The upregulation of IAA, ABA, GA_3_, and JA at stage 2 may promote male floral bud differentiation, whereas the upregulation of JA at stage 4 and ZT at stages 2 and 4 may promote hermaphroditic floral bud differentiation. Ninety-five and 183 TFs were differentially expressed at stages 2 and 4, respectively. *MYB*, *FAR1*, *bHLH*, *WRKY,* and *MADS* might play important roles in persimmon floral bud sex differentiation. Fifty-two and 54 DEGs at stages 2 and 4, respectively, participated in phytohormone biosynthesis and signaling pathways to regulate persimmon floral bud sex differentiation. Five metacaspases might perform vital functions in ‘Longyanyeshi 1’ male floral bud development by suppressing pistil primordia at stage 4. Integrated miRNA-mRNA analyses showed that several miRNAs involved in phytohormone biosynthesis and signaling pathways and floral organogenesis could regulate floral bud sex differentiation. This study laid an empirical foundation for ongoing investigations of floral bud sex differentiation in andromonoecious persimmon.

## Methods

### Plant materials

The ‘Longyanyeshi 1’ persimmon (*Diospyros kaki*) tree was a six-year-old seedling growing in Yuanyang County, Henan Province, China (34^o^55′18″ ~ 34^o^56′27″N, 113^o^46′14″ ~ 113^o^47′35″E) and it was cultivated from the seed of a wild *D. kaki* obtained in Longyan City, Fujian Province, Southeast China. The plant was an andromonoecious persimmon found by Fu and her research team [20]. All developmental stages of floral buds were proved to have stable horticultural characteristics during 3 years of field observation. The male and hermaphroditic floral buds were randomly collected every 3 d between March 28, 2018 and April 20, 2018 from the tree. Certain samples were fixed in FAA (formalin: glacial acetic acid: 50% (v/v) alcohol = 8:5:87, v/v). Others were immediately frozen in liquid nitrogen and stored at − 80 °C until phytohormone and RNA extraction.

### Paraffin section

Male and hermaphroditic floral buds fixed in FAA for 24 h were dehydrated in a graded ethanol series. They were then embedded in paraffin heated from 40 to 60 °C at an increment of 3 °C per 15 min. The samples were then immersed thrice in 60 °C paraffin for 2 h each time. Sections (5 μm thick) were prepared with a Leica RM2265 microtome (Leica Microsystems, Nussloch, Germany) and mounted on clean glass slides. The sections were deparaffinized and rehydrated in a graded xylol and ethanol series and stained overnight with hematoxylin. After staining with 1% eosin for 20 s, the sections were dehydrated in a graded ethanol and xylol series. The microslides were dried and mounted with cover slips and the stained sections were observed and photographed under a light microscope (Olympus, Tokyo, Japan).

### Phytohormone quantification by HPLC–ESI–MS/MS

Samples each weighing ~ 50 mg (FW) were transferred to 2-mL centrifuge tubes. The extraction and purification steps required for the determination of IAA, ABA, GA_3_, ZT, and JA were conducted according to the methods described by Pan [65].

High-performance liquid chromatography-electrospray ionization tandem-mass spectrometry (HPLC–ESI–MS/MS) in multiple-reaction monitoring (MRM) mode was used to quantitate the endogenous phytohormone levels. The HPLC–ESI–MS/MS consisted of an Agilent 1260 HPLC system (Agilent Technologies, Santa Clara, CA, USA) and an AB Qtrap 5500 triple quadrupole mass spectrometer (AB Sciex LLC, Framingham, MA, USA) with an electrospray ionization source. Samples were injected into an Agilent SB-C18 column (50 mm × 4.6 mm, 1.8 μm; Agilent Technologies, Santa Clara, CA, USA) and separated at a flow rate of 0.8 mL min^− 1^ with following mobile phases: acetonitrile (A) and distilled water with 0.1% acetic acid (B) for IAA, ABA, GA_3_, JA; and acetonitrile (A) and distilled water (B) for ZT. The HPLC gradient program and the multiple reaction monitoring (MRM) conditions used to quantify the phytohormones were adapted from Pan [65]. The injection volumes were 5.0 μL for IAA, ABA, GA_3_, and JA, and 1.0 μL for ZT.

Data acquisition and processing were performed in AB SCIEX Analyst v. 1.7 (AB Sciex LLC, Framingham, MA, USA). The content of each phytohormone was calculated as follows:

phytohormone content = (peak area of authentic phytohormone × amount of corresponding internal standard) / (peak area of corresponding internal standard × fresh weight of each sample).

### Total RNA extraction

Total RNA was isolated with TRIzol Reagent (Invitrogen, Carlsbad, CA, USA). RNA quality was tested with a Nano Drop 2000 spectrophotometer (Thermo Fisher Scientific, Wilmington, DE, USA) at 260 nm/280 nm (ratio > 2.0) and an Agilent 2100 bioanalyzer (Thermo Fisher Scientific, Waltham, MA, USA).

### Transcriptome sequencing and analysis

Sequencing libraries for mRNA were generated with an Illumina TruSeq RNA Sample Preparation Kit (Illumina, San Diego, CA, USA) following the manufacturer’s recommendations. The library was sequenced on a BGIseq500 platform (BGI Group, Shenzhen, Guangdong, China) and paired-end reads were generated. To obtain clean reads, the raw reads were filtered by removing low-quality reads and those containing adaptors and poly-N. Clean reads were assembled with Trinity v. 2.0.6 for de novo transcriptome assembly [66]. Unique genes were obtained with Tgicl v. 2.0.6 [67].

BLAST in the NR, NT, Swissprot, KEGG, KOG, Pfam, and GO databases was used for gene functional annotation. DEseq2 was used to detect differential mRNA expression (DEGs) (fold change ≥2; Q ≤ 0.001) [68]. Phyper was performed for the GO and KEGG enrichment analyses (Q ≤ 0.05).

### Small RNA sequencing and analysis

Sequencing libraries for small RNAs were generated with an Illumina TruSeq Small RNA Preparation Kit (Illumina, San Diego, CA, USA) following the manufacturer’s recommendations. The libraries were sequenced on a BGIseq500 platform (BGI Group, Shenzhen, Guangdong, China). After removing low-quality reads, the clean reads were mapped to the sRNA database (miRbase, siRNA, and snoRNA) with Bowtie v. 2.2.2.2.9 [69]. The miRA software was used to predict novel miRNA [70]. TAPIR, psRobot, and TargetFinder were used to predict target genes of miRNAs [71–73]. DEM analysis was performed using the DEGseq (Q ≤ 0.001; absolute value of Log2Ratio ≥ 1) [74]. To annotate gene functions, all target genes were aligned against the KEGG and GO databases. GO and KEGG enrichment analyses of target genes were performed using phyper (Q ≤ 0.05).

To determine the *OGI* expression level, small RNAs reads were mapped to the *D. lotus OGI* sequence [75], the *MeGI* sequence from the *D. oleifera* genome [76], and the ‘*Kali*’ sequence cloned from ‘Longyanyeshi 1’ persimmon (Fig. 4). Mapping was performed according to the method described by Akagi [5]*.* Expression levels were recorded as reads per million reads. Three biological replicates were analyzed per sample.

### Real-time quantitative polymerase chain reaction (RT-qPCR)

Total RNA for RNA-Seq and small RNA analysis was reverse-transcribed into cDNA with a TRUE-script 1st-Strand cDNA Synthesis Kit (Kemix, Beijing, China) and a Mir-X™ miRNA First-Strand Synthesis Kit (Takara, Dalian, China). The primers were used for RT-qPCR in a real-time system. Quantification of DEG and DEM expression was detected with TB Green™ Premix Ex Taq™ II (Tli RNaseH Plus) (Takara, Dalian, China) and a Mir-X miRNA RT-qPCR TB Green Kit (Takara, Dalian, China), respectively. The reaction conditions for the DEGs were 30 s at 95 °C followed by 40 cycles of 3 s at 95 °C and 30 s at 60 °C. The reaction conditions for the DEMs were 10 s at 95 °C followed by 40 cycles of 5 s at 95 °C and 20 s at 60 °C. Three technical replicates were performed for each mRNA and miRNA sample. *EF1-α* and *GAPDH* served as reference genes for mRNA RT-qPCR [77]. *U6* served as a reference gene for miRNA RT-qPCR. The relative expression values were obtained by the 2^-ΔΔCt^ method. The DEG and DEM primers for RT-qPCR are listed in Tables S8 and S9 (Additional file 9: Table S8 and Additional file 10: Table S9).

## Supplementary Information

**Additional file 1: Table S1.**

**Additional file 2: Table S2.**

**Additional file 3: Fig. S1.**

**Additional file 4: Table S3.**

**Additional file 5: Table S4.**

**Additional file 6: Table S5.**

**Additional file 7: Table S6.**

**Additional file 8: Table S7.**

**Additional file 9: Table S8.**

**Additional file 10: Table S9.**

## Data Availability

The materials of this study were provided by the Non-timber Forest Research and Development Center, Chinese Academy of Forestry. The raw sequencing data have submitted to the NCBI SRA database (PRJNA647029).

## Abbreviations

ABA: abscisic acid
DEG: differentially expressed gene
DEM: differentially expressed mRNA
ESI: electrospray ionization
FAA: formalin: acetic acid: alcohol
FR: forward repeat
FC: fold change
FPKM: fragments per kilobase per million
FW: fresh weight
GA_3_: gibberellic acid
GO: gene ontology
HPLC: high-performance liquid chromatography
IR: inverted repeat
IAA: indole-3-acetic acid
JA: jasmonic acid
KEGG: Kyoto Encyclopedia of Genes and Genomes
MiRNA: microRNA
MRM: multiple-reaction monitoring
MS/MS: tandem mass spectrometry
PCD: programmed cell death
RT-qPCR: real time quantitative polymerase chain reaction
TF: transcription factor
UTR: untranslated region
ZT: zeatin
