# Supplementary material for: Phytohormone and integrated mRNA and miRNA transcriptome analyses and differentiation of male between hermaphroditic floral buds of andromonoecious Diospyros kaki Thunb
Source: BMC Genomics. 2021 Mar 23;22:203. doi: 10.1186/s12864-021-07514-4 (PMC7986387; doi:10.1186/s12864-021-07514-4)
Supplement: Supplementary file 1 — Additional file 1: Table S1. [file 12864_2021_7514_MOESM1_ESM.docx]

**Table S1** Summary of data output quality of various libraries

| **Sample** | **Total Raw Reads (M)** | **Total Clean Reads (M)** | **Total Clean Bases(Gb)** | **Clean Reads Q20(%)** | **Clean Reads Q30(%)** | **Clean Reads Ratio(%)** |
| --- | --- | --- | --- | --- | --- | --- |
| HA1 | 47.33 | 44.42 | 6.66 | 96.84 | 88.10 | 93.87 |
| HA2 | 47.33 | 44.04 | 6.61 | 96.97 | 88.50 | 93.06 |
| HA3 | 47.33 | 44.32 | 6.65 | 96.85 | 88.13 | 93.65 |
| HB1 | 47.33 | 44.41 | 6.66 | 96.72 | 87.69 | 93.83 |
| HB2 | 45.57 | 42.65 | 6.40 | 96.78 | 87.90 | 93.59 |
| HB3 | 47.33 | 44.05 | 6.61 | 96.62 | 87.41 | 93.08 |
| MA1 | 47.33 | 44.40 | 6.66 | 96.77 | 87.82 | 93.82 |
| MA2 | 47.33 | 44.20 | 6.63 | 96.80 | 87.98 | 93.39 |
| MA3 | 45.57 | 42.75 | 6.41 | 96.86 | 88.20 | 93.81 |
| MB1 | 49.08 | 45.88 | 6.88 | 96.72 | 87.73 | 93.48 |
| MB2 | 47.33 | 44.00 | 6.60 | 96.62 | 87.40 | 92.96 |
| MB3 | 45.57 | 42.47 | 6.37 | 96.69 | 87.61 | 93.19 |

Q20: The percentage of bases with a Phred value > 20

Q30: The percentage of bases with a Phred value > 30
