# Supplementary material for: Phytohormone and integrated mRNA and miRNA transcriptome analyses and differentiation of male between hermaphroditic floral buds of andromonoecious Diospyros kaki Thunb
Source: BMC Genomics. 2021 Mar 23;22:203. doi: 10.1186/s12864-021-07514-4 (PMC7986387; doi:10.1186/s12864-021-07514-4)
Supplement: Supplementary file 2 — Additional file 2: Table S2. [file 12864_2021_7514_MOESM2_ESM.docx]

**Table S2** Summary of assembly results of various libraries

| **Sample** | **Total Number** | **Total Length** | **Mean Length** | **N50** | **N70** | **N90** | **GC(%)** |
| --- | --- | --- | --- | --- | --- | --- | --- |
| HA1 | 45721 | 48349301 | 1057 | 1657 | 1095 | 467 | 43.91 |
| HA2 | 46198 | 48305252 | 1045 | 1654 | 1089 | 453 | 43.96 |
| HA3 | 44584 | 47952264 | 1075 | 1670 | 1117 | 481 | 44.04 |
| HB1 | 50430 | 51910826 | 1029 | 1623 | 1054 | 444 | 43.52 |
| HB2 | 47621 | 50172484 | 1053 | 1653 | 1094 | 461 | 43.75 |
| HB3 | 47426 | 49643309 | 1046 | 1639 | 1080 | 459 | 43.81 |
| MA1 | 45919 | 48392567 | 1053 | 1660 | 1096 | 458 | 43.91 |
| MA2 | 45816 | 47573956 | 1038 | 1637 | 1073 | 449 | 43.97 |
| MA3 | 44998 | 47286312 | 1050 | 1646 | 1086 | 458 | 44.00 |
| MB1 | 50614 | 53027364 | 1047 | 1640 | 1074 | 461 | 43.59 |
| MB2 | 49034 | 51374575 | 1047 | 1647 | 1084 | 456 | 43.73 |
| MB3 | 46727 | 49562830 | 1060 | 1648 | 1095 | 471 | 43.89 |
| All-Unigene | 82910 | 114119802 | 1376 | 2071 | 1439 | 691 | 43.03 |

N50: a weighted median statistic that 50% of the total length is contained in transcripts great than or equal to this value

N70: a weighted median statistic that 70% of the total length is contained in transcripts great than or equal to this value

N90: a weighted median statistic that 90% of the total length is contained in transcripts great than or equal to this value
