# Supplementary figures and images for: Phytohormone and integrated mRNA and miRNA transcriptome analyses and differentiation of male between hermaphroditic floral buds of andromonoecious Diospyros kaki Thunb
Source: BMC Genomics. 2021 Mar 23;22:203. doi: 10.1186/s12864-021-07514-4 (PMC7986387; doi:10.1186/s12864-021-07514-4)

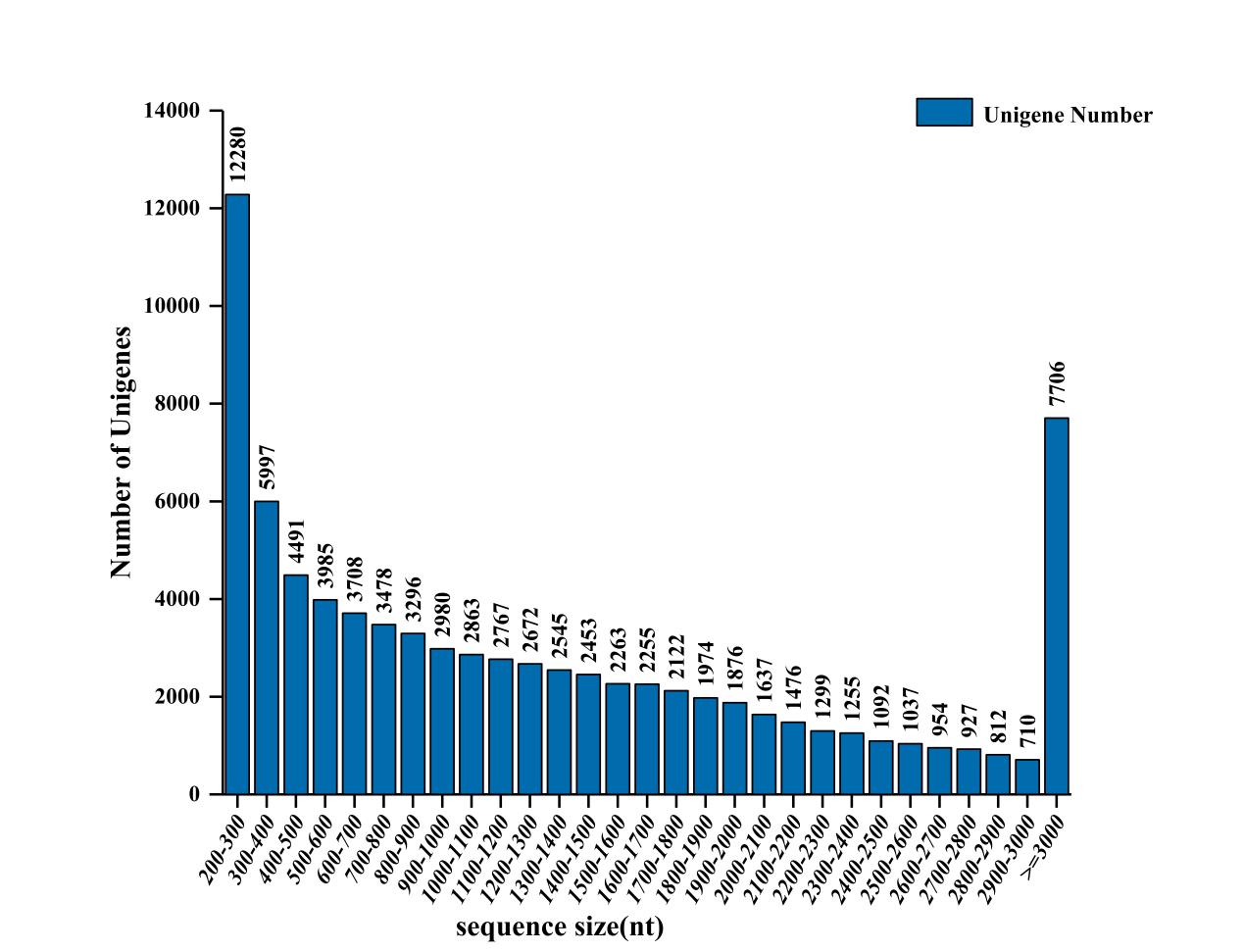


**Fig. S1** Distribution size of de novo assembled unigenes

Supplement: Supplementary file 3 — Additional file 3: Fig. S1. [file 12864_2021_7514_MOESM3_ESM.docx]
