# Supplementary material for: Phytohormone and integrated mRNA and miRNA transcriptome analyses and differentiation of male between hermaphroditic floral buds of andromonoecious Diospyros kaki Thunb
Source: BMC Genomics. 2021 Mar 23;22:203. doi: 10.1186/s12864-021-07514-4 (PMC7986387; doi:10.1186/s12864-021-07514-4)
Supplement: Supplementary file 4 — Additional file 4: Table S3. [file 12864_2021_7514_MOESM4_ESM.docx]

**Table S3** Numbers and frequencies of unigenes annotated in public databases

| **Values** | **Total** | **NR** | **NT** | **Swissprot** | **KEGG** | **KOG** | **Pfam** | **GO** | **Intersection** | **Overall** |
| --- | --- | --- | --- | --- | --- | --- | --- | --- | --- | --- |
| Number | 82,910 | 62,021 | 50,224 | 46,639 | 49,790 | 49,614 | 47,121 | 34,735 | 20,234 | 64,355 |
| Percentage | 100% | 74.81% | 60.58% | 56.25% | 60.05% | 59.84% | 56.83% | 41.89% | 24.40% | 77.62% |
