# Supplementary material for: Phytohormone and integrated mRNA and miRNA transcriptome analyses and differentiation of male between hermaphroditic floral buds of andromonoecious Diospyros kaki Thunb
Source: BMC Genomics. 2021 Mar 23;22:203. doi: 10.1186/s12864-021-07514-4 (PMC7986387; doi:10.1186/s12864-021-07514-4)
Supplement: Supplementary file 7 — Additional file 7: Table S6. [file 12864_2021_7514_MOESM7_ESM.docx]

**Table S6** Overview of reads for small RNA-seq from raw data to high quality reads, and quality filtering

| **Sample name** | **Raw tag count** | **Low quallity tag count** | **Invalid adapter tag count** | **PolyA tag count** | **Short valid length tag** | **Clean tag count** | **Q20 of clean tag (%)** | **Percentage of clean tag(%)** |
| --- | --- | --- | --- | --- | --- | --- | --- | --- |
| HA1 | 29270029 | 467473 | 425501 | 1505 | 344408 | 28031142 | 99.3 | 95.77 |
| HA2 | 30191442 | 515466 | 614664 | 2344 | 356857 | 28702111 | 99.4 | 95.07 |
| HA3 | 29712355 | 686173 | 457027 | 1256 | 756428 | 27811471 | 99.3 | 93.6 |
| HB1 | 29312426 | 626032 | 465413 | 1498 | 1147834 | 27071649 | 99.4 | 92.36 |
| HB2 | 29842347 | 647553 | 495566 | 1399 | 354264 | 28343565 | 99.4 | 94.98 |
| HB3 | 29315312 | 484593 | 406972 | 1181 | 227943 | 28194623 | 99.4 | 96.18 |
| MA1 | 29419504 | 527641 | 570739 | 2125 | 383047 | 27935952 | 99.3 | 94.96 |
| MA2 | 28937665 | 506979 | 531229 | 1407 | 666373 | 27231677 | 99.3 | 94.1 |
| MA3 | 28453279 | 600086 | 416209 | 1379 | 334940 | 27100665 | 99.2 | 95.25 |
| MB1 | 30016434 | 459423 | 441893 | 1018 | 736691 | 28377409 | 99.3 | 94.54 |
| MB2 | 29838592 | 641368 | 461933 | 772 | 541390 | 28193129 | 99.3 | 94.49 |
| MB3 | 29517270 | 726662 | 381816 | 630 | 705749 | 27702413 | 99.3 | 93.85 |
