# Supplementary material for: Phytohormone and integrated mRNA and miRNA transcriptome analyses and differentiation of male between hermaphroditic floral buds of andromonoecious Diospyros kaki Thunb
Source: BMC Genomics. 2021 Mar 23;22:203. doi: 10.1186/s12864-021-07514-4 (PMC7986387; doi:10.1186/s12864-021-07514-4)
Supplement: Supplementary file 9 — Additional file 9: Table S8. [file 12864_2021_7514_MOESM9_ESM.docx]

**Table S8** The primers of genes used in RT-qPCR analyses.

| **Stage** | **Gene ID** | **Forward sequence (5’-3’)** | **Reverse sequence (5’-3’)** | **Amplicon length (bp)** |
| --- | --- | --- | --- | --- |
|  | *EF1-α* (reference gene) | CAAGAGGCCTTCAGACAAGC | ACCATGCCAGGTTTGAGAAC | 117 |
|  | *GAPDH* (reference gene) | AGCTCTTCCACCTCTCCAGT | TGCTAGCTGCACAACCAACT | 157 |
| Stage 2 | CL6110.Contig2_All (*MeGI*) | TGGGACGACACCAAGGAGAA | CCATCGAGCCCTTCTATTCTGA | 248 |
|  | CL1306.Contig2_All (*ARR*) | TGCCTGGAATGACTGGTTATGA | GATGAGATGGACGGCTGCG | 307 |
|  | CL6188.Contig1_All (*ARR*) | ATAGATGTTTGGAGGAAGGGGC | TTGGCAACAACGATAAAAAGGC | 189 |
|  | Unigene9386_All (*CYP735A*) | CTCTTCCCCCATTTCCTCGTCT | ACAATGTGCCTCTGATGATGCC | 233 |
|  | Unigene16479_All (*MADS*) | ACCAACCGCCAAGTTACATTCT | CGTGGGGCATGTAGTGGAAGTA | 204 |
|  | Unigene18753_All (*MADS*) | AGAATGACACGAGCAGGCAAGT | CTTTTGCGTGCTTGCGATACC | 189 |
|  | CL1678.Contig7_All (*TCP2*) | GTGGAATGGCTACTCAAGGCTG | GCTTTTATGCGACGCTTAGACC | 281 |
|  | CL9066.Contig2_All (*ACO*) | TCAACGATGCCTGTGAGAACTG | AGATCGGGGAGTTCTGAGAGGT | 247 |
|  | CL3175.Contig1_All (*JAZ*) | TGGGAACCAACAGAAACAGCAG | AAGGAGAAGTAGCCGAAAGCCG | 290 |
|  | CL6576.Contig1_All (*JAZ*) | CGAAGTTGAATCCTGACCTCCT | AACCATAAGAAATGCTCCCGAA | 180 |
|  | CL3250.Contig22_All (*DELLA*) | ATGAGGTTGGCTGGAGAAAGGT | CAAATTGCTGGTAGCCCACTTT | 178 |
| Stage 4 | CL6110.Contig2_All (*MeGI*) | TGGGACGACACCAAGGAGAA | CCATCGAGCCCTTCTATTCTGA | 248 |
|  | CL1306.Contig2_All (*ARR*) | TGCCTGGAATGACTGGTTATGA | GATGAGATGGACGGCTGCG | 307 |
|  | CL6188.Contig1_All (*ARR*) | ATAGATGTTTGGAGGAAGGGGC | TTGGCAACAACGATAAAAAGGC | 189 |
|  | CL9066.Contig2_All (*ACO*) | TCAACGATGCCTGTGAGAACTG | AGATCGGGGAGTTCTGAGAGGT | 247 |
|  | CL309.Contig7_All (*MADS*) | CGGTTCTTTGTGATGCTGAGGT | GAGATTCCTTTGGGTTCGTTGA | 233 |
|  | CL6521.Contig6_All (*MADS*) | AGAGAATCCAACGAACAGGCAG | TCGTAGTGCGTGTTCCATAGGTC | 222 |
