# Supplementary material for: Phytohormone and integrated mRNA and miRNA transcriptome analyses and differentiation of male between hermaphroditic floral buds of andromonoecious Diospyros kaki Thunb
Source: BMC Genomics. 2021 Mar 23;22:203. doi: 10.1186/s12864-021-07514-4 (PMC7986387; doi:10.1186/s12864-021-07514-4)
Supplement: Supplementary file 10 — Additional file 10: Table S9. [file 12864_2021_7514_MOESM10_ESM.docx]

**Table S9** The primers of miRNAs used in RT-qPCR analyses.

| **Stage** | **miRNA ID** | **Forward primer** |
| --- | --- | --- |
| Stage 2 | miR157d-3p | GCTCTCTATGCTTCTGTCATC |
|  | novel_mir27 | GCTTTACTTCGGAGCGGGAGTACTCGA |
|  | miR157a-5p | TTGACAGAAGATAGAGAGCAC |
|  | miR157d | TGACAGAAGATAGAGAGCAC |
|  | miR169e_3 | AGCCAAGGATGACTTGCCGG |
|  | miR319a | CTTGGACTGAAGGGAGCTCC |
|  | miR390e | AGCTCAGGAGGGATAGCGCC |
| Stage 4 | miR166m_2 | CGGACCAGGCTTCATTCCCC |
|  | miR171a_3 | TGATTGAGCCGTGCCAATAT |
|  | novel_mir1 | GTGGATTGGACATTTAGTTTGC |
|  | miR169v_1 | CAGCCAAGGATGACTTGCC |
|  | miR390e | AGCTCAGGAGGGATAGCGCC |
